# HIV Associated Neurocognitive Disorders (HAND) in Malawian Adults and Effect on Adherence to Combination Anti-Retroviral Therapy: A Cross Sectional Study

**DOI:** 10.1371/journal.pone.0098962

**Published:** 2014-06-10

**Authors:** Christine M. Kelly, Joep J. van Oosterhout, Chisomo Ngwalo, Robert C. Stewart, Laura Benjamin, Kevin R. Robertson, Saye Khoo, Theresa J. Allain, Tom Solomon

**Affiliations:** 1 Brain Infection Group, Institute of Infection and Global Health, University of Liverpool, Liverpool, United Kingdom; 2 Malawi-Liverpool-Wellcome Clinical Research Programme, Queen Elizabeth Central Hospital, College of Medicine, Blantyre, Malawi; 3 Department of Medicine, College of Medicine, Blantyre, Malawi; 4 Department of Mental Health, College of Medicine, Blantyre, Malawi; 5 Department of Neurology, University of North Carolina, Chapel Hill, North Carolina, United States of America; 6 HIV Pharmacology Group, Institute of Translational Medicine, University of Liverpool, Liverpool, United Kingdom; 7 Dignitas International, Zomba, Malawi; 8 Walton Centre NHS Foundation Trust, Liverpool, United Kingdom; 9 Health Protection Research Unit in Emerging and Zoonotic Infections, Liverpool, United Kingdom; Imperial College London, United Kingdom

## Abstract

**Background:**

Little is known about the prevalence and burden of HIV associated neurocognitive disorder (HAND) among patients on combination antiretroviral therapy (cART) in sub-Saharan Africa. We estimated the prevalence of HAND in adult Malawians on cART and investigated the relationship between HAND and adherence to cART.

**Methods:**

HIV positive adults in Blantyre, Malawi underwent a full medical history, neurocognitive test battery, depression score, Karnofsky Performance Score and adherence assessment. The Frascati criteria were used to diagnose HAND and the Global Deficit Score (GDS) was also assessed. Blood was drawn for CD4 count and plasma nevirapine and efavirenz concentrations. HIV negative adults were recruited from the HIV testing clinic to provide normative scores for the neurocognitive battery.

**Results:**

One hundred and six HIV positive patients, with median (range) age 39 (18–71) years, 73% female and median (range) CD4 count 323.5 (68–1039) cells/µl were studied. Symptomatic neurocognitive impairment was present in 15% (12% mild neurocognitive disorder [MND], 3% HIV associated dementia [HAD]). A further 55% fulfilled Frascati criteria for asymptomatic neurocognitive impairment (ANI); however factors other than neurocognitive impairment could have confounded this estimate. Neither the symptomatic (MND and HAD) nor asymptomatic (ANI) forms of HAND were associated with subtherapeutic nevirapine/efavirenz concentrations, adjusted odds ratio 1.44 (CI. 0.234, 8.798; p = 0.696) and aOR 0.577 (CI. 0.09, 3.605; p = 0.556) respectively. All patients with subtherapeutic nevirapine/efavirenz levels had a GDS of less than 0.6, consistent with normal neurocognition.

**Discussion/Conclusion:**

Fifteen percent of adult Malawians on cART had a diagnosis of MND or HAD. Subtherapeutic drug concentrations were found exclusively in patients with normal neurocognitive function suggesting HAND did not affect cART adherence. Further study of HAND requires more robust locally derived normative neurocognitive values and determination of the clinical relevance of ANI.

## Introduction

Prevalence estimates for HIV associated neurocognitive disorder (HAND) in patients on combination anti-retroviral therapy (cART) range from 19% to 52% in high resource settings [1]–[3], and from 14% to 64% in low resource settings [4]–[13]. However, the consequences of neurocognitive impairment in sub-Saharan Africa, the region with the highest global burden of HIV, remain unclear. The detrimental effects of HAND on an individual's ability to work, interact socially and adhere to medications have been clearly demonstrated in high resource settings [14]–[21]. Given that the proportion of HAND in patients on cART in sub-Saharan Africa appears to be high, and that 20 million people are projected to be on cART in this region in the next 5 years, it is essential that the burden of persistent neurocognitive impairment is better established [5], [22].

It has long been recognised that the direct effects of HIV on the brain lead to neurocognitive impairment and, if left untreated, progress to HIV associated Dementia (HAD) [23]. Although HAD is usually reversible with cART, and so has become much less common since the advent of cART, mild and moderate forms of neurocognitive impairment remain common so that prevalence rates have actually increased in patients on cART [24]. However, prevalence rates vary due to several factors, including a large variation in the instruments used for testing neurocognitive impairment; strong effects of age, gender and educational level on neurocognitive performance; and the role played by co-morbidities and prior central nervous system (CNS) disease, including opportunistic infections [25].

**Figure 1:**
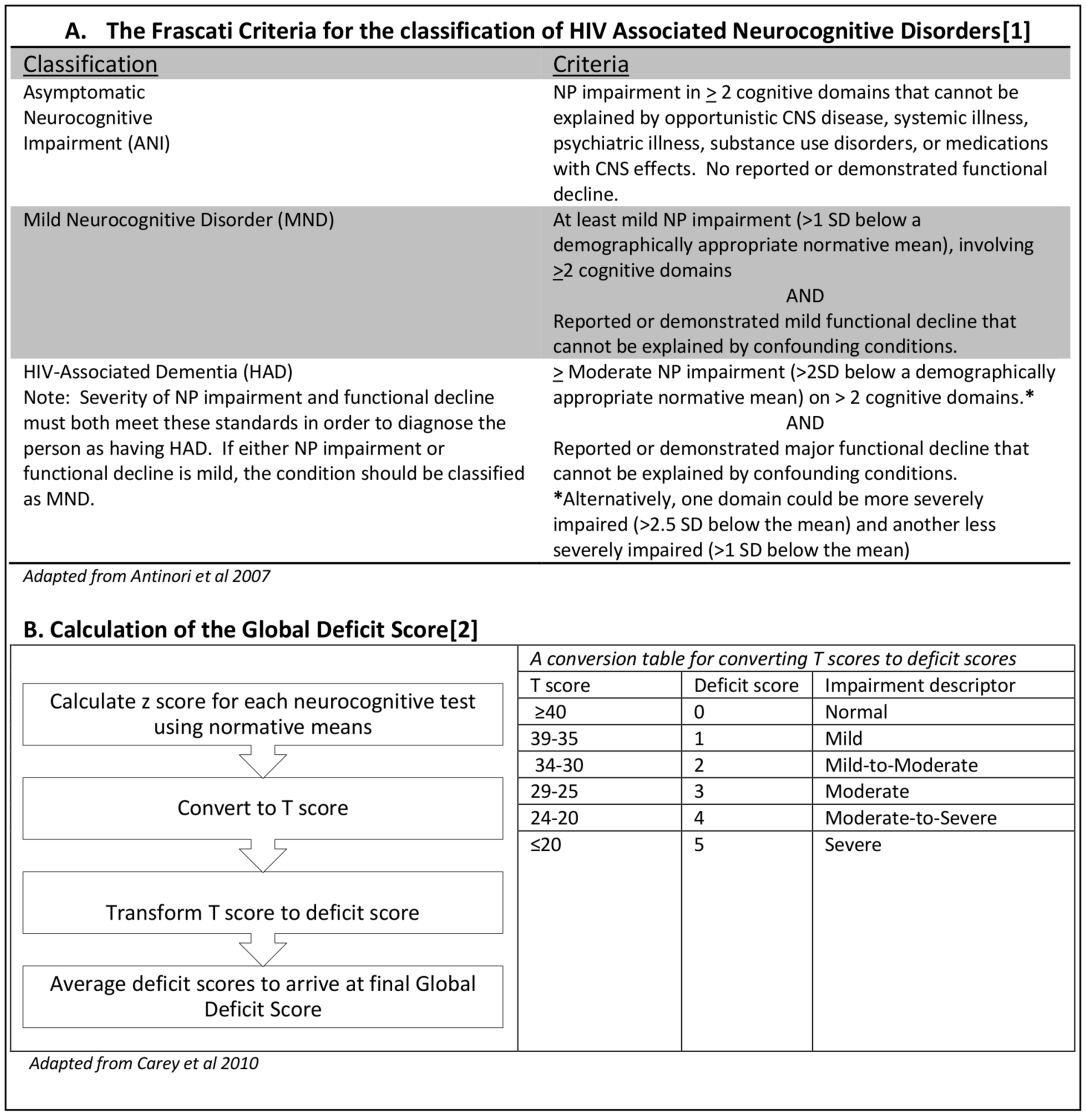
Comparisons of the Frascati Criteria and Global Deficit Scores to define neurocognitive impairment in HIV.

Neurocognitive impairment is demonstrated by abnormalities on neurocognitive test batteries and can be quantified using scoring systems based on z scores, such as the Global Deficit Score (GDS)[26]. The Frascati criteria [27] proposed a system for categorising neurocognitive impairment in HIV into a spectrum of disorders. These are referred to as HAND and range from asymptomatic neurocognitive disorder (ANI), to mild neurocognitive disorder (MND), to HAD. The criteria are distinct from other scoring systems such as the GDS not only in how neurocognitive impairment is classified but also in the inclusion of an assessment of the clinical impact of that impairment. The thresholds used to define neurocognitive impairment in these criteria are outlined in detail in Antinori et al, 2007 [27], and are based on standard deviations around normative means. These two systems for classifying neurocognitive impairment in HIV are described in detail in Figure 1.

In sub-Saharan Africa the diagnosis has thus far been based largely on the International HIV Dementia Scale (IHDS) [10], which was designed to quickly identify patients at risk of HAD but not to diagnose HAND. It has not performed consistently in patients on cART [7], [8], [28]. Neurocognitive test batteries are considered the ‘gold standard’ for diagnosing HIV related neurocognitive impairment in Western settings, but only two cohorts from sub-Saharan Africa have reported prevalence of HAND based on formal neurocognitive testing [5], [29].

We aimed to establish normative values for a neurocognitive test battery and to estimate the prevalence of HAND in adult Malawians on cART. In order to begin to assess the consequences of HAND in sub-Saharan Africa, we also aimed to characterise the relationship between HAND and adherence to cART as measured by plasma concentrations of efavirenz or nevirapine.

## Methods

### Ethics statement

The study was approved by the College of Medicine Research and Ethics Committee (registration number P.07/11/1104). All participants provided written informed consent.

### Patients and setting

This cross sectional study was conducted in the cART clinic at Queen Elizabeth Central Hospital (QECH), Blantyre, Malawi from August 2011 until March 2012. Patients were approached consecutively and assessed for eligibility for enrolment. The inclusion criteria were: over 18 years of age and on cART for at least 3 months. The exclusion criteria were: Glasgow Coma Score<15, acute illness or fever, history of neuro-denegerative disease, physical disability impairing the capacity to carry out neurocognitive tests). Those who met study criteria were screened with the IHDS. It would not have been feasible within the constraints of this study to perform full neurocognitive testing on all patients approached. In order to obtain an adequate number of patients with HAND to make comparisons on adherence, the patients were recruited on a one to one basis according to whether they had a positive or negative IHDS. All those scoring positive on the IHDS (<10.5) were invited for enrolment as well as an equal number of patients screening negatively (the first patient who screened negative after one positive patient was enrolled). Patients then underwent a full medical history, adherence assessment, depression assessment, neurocognitive assessment, Karnofsky Performance Score [30], neurological examination and venepuncture. Occupation level was determined on a 5 point scale where 0 = unemployed and 4 = highly skilled worker. Schooling was assessed as number of years of education successfully completed.

Ideally, a diagnosis of HAND should be made after the exclusion of other neurological co-morbidities. In order to give an indication of whether patients had another explanation for the clinical picture, five patients with the worst performance on neurocognitive tests were chosen to undergo a lumbar puncture and brain magnetic resonance imaging (MRI).

HIV negative adults were identified from the adjacent voluntary counselling and testing (VCT) clinic. Participants were frequency matched for age category (< or ≥35 years) and education level category (≤ or >7 years) and were subject to the same inclusion and exclusion criteria as HIV positive participants.

### Neuropsychological test battery

The test battery was chosen so as to be comprehensive and applicable in a low resource setting. It included all three tests from the ACTG 5199 study [22] in order to be comparable to other data collected in Malawi, as well as additional tests from the World Health Organisation (WHO) neuropsychological battery and the HIV Neurobehavioural Research Centre (HNRC) international test battery [29]. We used the Hopkins verbal learning test revised (HVLT-R) to assess learning and memory, timed gait for gross motor skills, finger tapping and grooved pegboard for fine motor skills, semantic verbal fluency for language fluency, colour trails 1 and WAIS digit symbol for attention and speed of information processing and finally colour trails 2 for set shifting and response inhibition. A version of HVLT-R which had been previously translated into Chichewa and back translated for verification was used [22]. Participants needed to be able to count to 25 before participating in the colour trails. The tests were administered by the study clinician who underwent training in administration of the tests prior to study onset. The tests were translated into Chichewa for all participants by a translator who also underwent training in the administration of the tests.

### Other assessment tools

The self-reporting questionnaire – 20 (SRQ-20) was used to assess for symptoms of depression and anxiety. This tool has previously been translated into Chichewa, back translated into English, and validated for use in Malawi [31]. Both the study clinician and translator underwent training in the administration of the SRQ-20 by a psychiatrist prior to study onset.

The average percentage pill count was measured to provide a subjective score of adherence. Pill counts are performed routinely at every patient refill and recorded on a computerised database. The number of pills taken was calculated as the number dispensed minus the number counted. This was then divided by the number of doses the patient was expected to have taken (number of daily doses multiplied by days since refill). The percentages of the preceding 3 months were averaged to give the average percentage pill count.

### Laboratory tests

CD4 counts were quantified from whole blood using an automated FACS count machine (Becton-Dickinson, Belgium).

Plasma was stored at −25° and transported on dry ice to Liverpool, UK for determination of nevirapine (NVP) and efavirenz (EFV) concentrations using liquid chromatography and mass spectrometry, as previously described [32]. Samples were heat denatured prior to analysis. Drug concentrations below the minimal effective concentration (MEC) were classified as suboptimal and were defined as 1000 ng/mL for EFV and 3000 ng/mL for NVP. The upper toxicity threshold for EFV is 4000 ng/mL whilst there is no consensus on the acceptable upper limit for NVP [33].

### Lumbar puncture and brain MRI

Cerebrospinal fluid (CSF) samples were processed for gram stain, cell counts, protein and glucose, cryptococcal antigen, stain for acid and alcohol fast bacilli (AAFB) and tuberculosis (TB) culture. MRI scans were performed at the MRI facility at QECH.

### Statistical analyses

Data were entered onto the REDcap electronic data capture system (Vanderbilt 2013) [34] using double data entry. As the prevalence of subtherapeutic drug concentrations was unknown in our population, a convenience sample was taken.

Normative scores for all the neuropsychological tests were calculated by using the mean and standard deviations from HIV negative participants. These were then used to calculate z scores for the HIV positive patients. For the diagnosis of HAND the Frascati criteria were applied [27]. Z scores were used to determine if a patient was neurocognitively impaired according to Frascati criteria. A patient was then classified as having MND if they complained of symptoms thought to be related to neurocognitive impairment and as having HAD if they had a Karnofsky performance score of less than 80% due to neurocognitive symptoms. To provide a continuous measurement of purely neurocognitive performance, a global deficit score (GDS) [26] was calculated by finding the average of all the z scores for each of 13 neurocognitive tests.

The Mann Whitney-U test and chi square test were used to analyse non-parametric and categorical data respectively. Models to determine associations of patient characteristics with subtherapeutic NNRTI drug levels and with presence of HAND were constructed using binomial and linear regression. HAND was divided into the combination of MND or HAD as one outcome measure and ANI as another. Variables included in the final analysis were age, gender, schooling, SRQ score, number of cART regimens, pill count and NNRTI. All statistical analysis was conducted on SPSS Statistics Version 20.

## Results

### Recruitment

**Figure 2:**
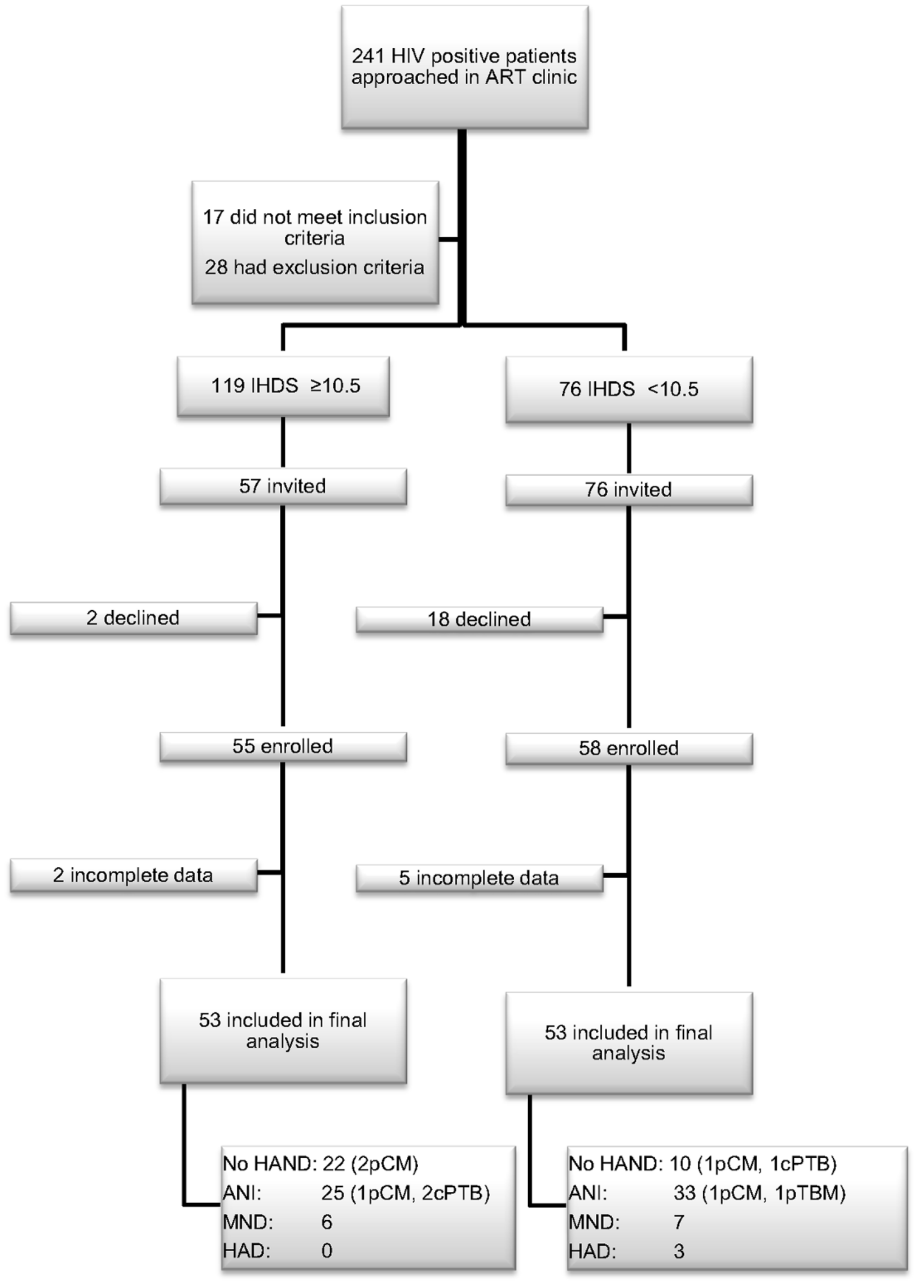
Summary of patient screening and recruitment. ART: anti-retroviral therapy, HIV: human immunodeficiency virus, VCT: voluntary testing and counselling, IHDS: International HIV Dementia Scale, HAND: HIV associated neurocognitive disorder, ANI: asymptomatic neurocognitive impairment, MND: minor neurocognitive disorder, HAD: HIV associated dementia, pCM: past history of cryptococcal meningitis, pTBM: past history of tuberculous meningitis, cPTB: current pulmonary tuberculosis.

One hundred and ninety five patients with HIV were screened with the IHDS, 113 were enrolled and 106 were included in the final analysis (figure 2). A further 103 consecutive HIV negative participants were recruited from the VCT clinic.

### Characteristics of participants

For the HIV positive group the median (range) age was 39 (18–71) years, 73% were female, 70% had 7 years or less of education and 86% were either unemployed or an unskilled worker. Three patients were receiving treatment for pulmonary TB at the time of the study and six patients had a past history of CNS infection; five cryptococcal meningitis, one TB meningitis (figure 2). Only one patient had a history of smoking or alcohol use and four patients had a history of cardiovascular disease (three hypertension, one angina and one mitral stenosis with heart failure). Patients had been on cART for a median of 33 months (range 3–136) and 44% had experienced at least one change of cART regimen. The median pill count was 99% (range 67.5%–100%). A score of ≥8 on the SRQ-20 was found in 41% of patients with HIV and in 24% of those without.

**Figure 3:**
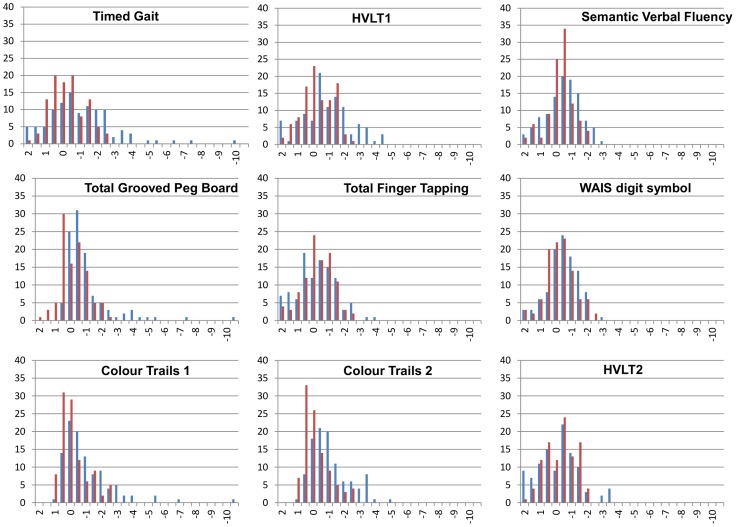
Distributions of neurocognitive test results according to HIV status in 209 Malawians adults. The horizontal axis shows the z score calculated using the standard deviation around the HIV negative mean. The vertical axis shows the frequency of patients achieving each z score. The blue bars represent HIV positive patients and the red bars represent HIV negative patients. HVLT  =  Hopkins Verbal Learning Test.

The HIV negative group provided normative values for neurocognitive tests. The median age was 34.5 years (range 18–60), 53% were male, 49% had 7 years of education or less and 74% were either unemployed or an unskilled worker. Baseline characteristics of patients with and without HIV are compared in table 1 and normative values for neurocognitive tests are shown in table 2. Of note, HIV negative participants were significantly more likely to have higher educational attainment (P = 0.001) and higher occupational level (P = 0.028), and less likely to have anxiety or depression (as judged by SRQ-20 score; P = 0.002). Forty one percent of HIV negative participants fulfilled the Frascati criteria for neurocognitive impairment based on the neurocognitive test battery. Distributions of neurocognitive test values for HIV negative and positive patients are shown in figure 3. None of the HIV negative participants had a z score of -3 or less on any neurocognitive test but HIV positive patients showed a large range of extreme abnormal z scores. Thirty two (31%) HIV positive patients had 2 impaired domains compared to 13 (13%) HIV negative patients.

**Table 1:** Baseline characteristics of 209 Malawian adults with and without HIV.

Baseline characteristic	HIV positive (n = 106)*	HIV negative (n = 103)	P value
**Age (range)**	39 (18–71)	34.5 (18–60)	<0.001
**Number Female (%)**	77 (73)	55 (53)	0.007
**Median number of years of school completed (range)**	7 (0–21)	10.5 (0–21)	0.001
**Number with occupational level less than 3 (%)**	102 (96)	76 (74)	0.028
**Number with SRQ-20 score ≤8 (%)**	41 (40%)	24 (23%)	0.002
**Median BMI (range)**	22.5 (15.9–47.4)	-	-
**Median pre-cART CD4 count cells/uL (range)**	182 (2–802)*		
**Median study CD4 count cells/uL (range)**	323.5 (68–1039)		
**Median duration of cART in months (range)**	33 (3–136)	-	-
**Median number of cART regimens (range)**	1 (1–3)	-	-

HIV: human immunodeficiency virus, BMI: Body mass index, WHO: World Health Organisation, cART: combination anti-retroviral therapy. *Pre-ART CD4 counts were only available for 71 (67%) HIV positive patients.

**Table 2:** Normative values of a neurocognitive battery from 103 HIV negative Malawian adults according to age ≥35 years or <35 years and ≥7 years or <7 years of schooling.

	age ≤35 years school ≤7 years		age ≤35 years school >7 years		age >35 years school ≤7 years		age >35 year school >7 years	
	Av	SD	Av	SD	Av	SD	Av	SD
**TG (secs)**	10.3	1.5	10.3	1.4	11.0	1.2	11.0	0.9
**HVLT, TR**	16.6	2.7	18.9	3.6	17.8	2.8	19.9	3.6
**GPB, D (secs)**	71.8	19.2	68.3	17.8	77.2	54.0	47.7	13.1
**GPB, ND (secs)**	88.4	22.0	81.9	13.1	99.0	49.0	69.2	16.9
**SVF**	18.1	4.4	21.5	5.1	19.0	4.3	21.1	3.5
**FT, D**	31.0	6.2	32.2	7.5	31.3	5.1	34.1	5.6
**FT, ND**	30.7	6.0	32.4	7.2	29.6	5.4	32.5	7.2
**CT1 (secs)**	77.8	38.4	61.6	25.2	66.6	30.8	44.9	11.1
**CT2 (secs)**	162.4	80.5	122.9	48.7	140.5	78.3	94.7	28.1
**WAIS DST**	31.4	10.3	45.2	13.1	30.5	14.0	44.5	9.4
**HVLT DR (%)**	76.4	25.3	85.0	22.5	76.0	24.2	76.4	17.1
**HVLT RDI**	10.0	2.1	10.8	1.2	9.3	2.5	11.5	0.8

Where results are a measurement of time, units are shown in seconds (secs). Av =  mean, sd = standard deviation, TG =  Timed Gait, HVLT = Hopkins verbal learning test, TR = total recall, GPB  =  grooved pegboard, D = dominant, ND = non-dominant, SVF = semantic verbal fluency, CT = colour trails, WAIS DST = WAIS digit symbol test, DR = delayed recall, RDI = recognition discrimination index.

### Prevalence of HAND

HAD was present in 3% and minor cognitive and motor disorder in 12%. Fifty five percent of patients met Frascati criteria for asymptomatic neurocognitive impairment. All 3 patients with a final diagnosis of HAD scored less than 8 on the IHDS. Seventy five percent of patients with a positive IHDS met criteria for MND or ANI compared to 58% of those with a negative IHDS. For patients with a final diagnosis of HAD, MND, ANI and no HAND, the median (range) IHDS scores were 7.5 (6.5–8), 10 (8–12), 10 (8–12), 11 (7.5–11) respectively. The median number of impaired neurocognitive tests was 2 (range 0–13) and the median Karnofsky Performance Score was 100% (range 70–100). The median SRQ-20 score was 5 out of 20 (range 0–16) and 32% of patients scored SRQ ≥8 indicating probable depression.

For those patients who underwent lumbar puncture, the CSF was unremarkable apart from a mildly raised protein in one patient (0.49 g/L). Gram stain, stain for acid fast bacilli, cryptococcal antigen and TB culture were negative in all 5 cases. HIV-1 RNA was undetectable in all CSF samples. One patient had detectable plasma viral load (150 copies/ml). This patient had started cART 4 months before study entry. Five patients demonstrated findings consistent with HAD on brain MRI; 3 out of the 5 patients had diffuse leuco-encephalopathy with ventricular dilatation and the remaining 2 patients had diffuse atrophy only). No patients had any other cause for neurocognitive impairment elucidated on brain MRI.

### Adherence and relationship to HAND

Eighty six patients were taking nevirapine based cART and the other 20 were on an efavirenz based regimen. Concurrent medications were recorded for all patients: 4 patients were on medications that could reduce plasma NNRTI concentrations and 3 were on medications that could increase them. All patients were on co-trimoxazole preventive therapy.

**Figure 4:**
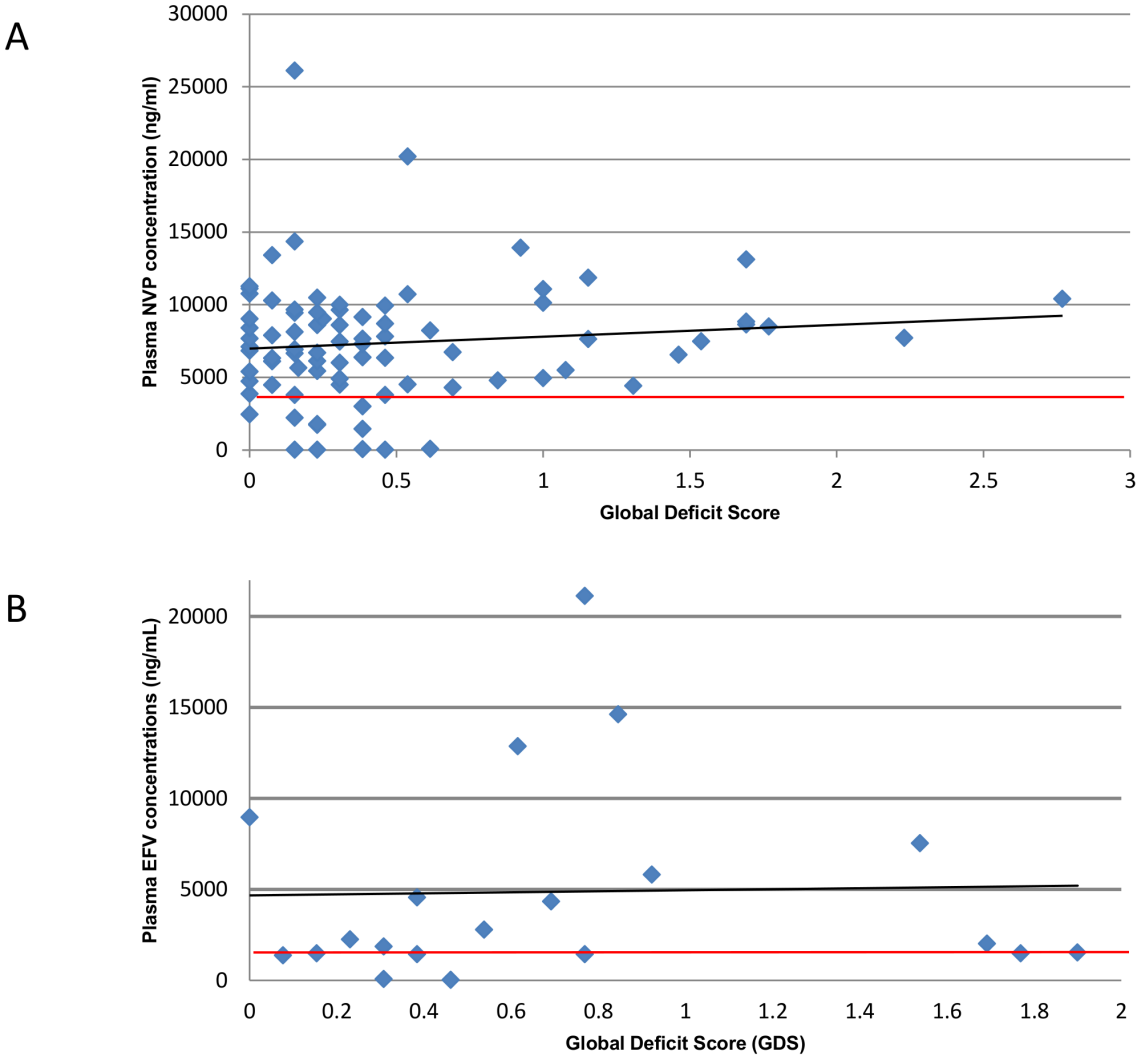
Plasma NNRTI concentrations according to Global Deficit Score (GDS)* in 103 HIV positive Malawian adults. (A. nevirpaine B. efavirenz). The black line represent the best fit linear trend and the red line represents the Minimum Effectivenes Concentration (MEC). *A Global Deficit Score (GDS) of between 0.5 and 1 represents borderline/mild neurocognitive impairment.

Associations of variables with subtherapeutic NNRTI levels are outlined in table 3. Pill count, was independently associated with a decreased adjusted risk of subtherapeutic drug concentrations (for each percentage rise in average pill count: adjusted OR 0.955, CI 0.914–0.997, p0.036). Of note, all patients with subtherapeutic drug concentrations had a GDS of less than 0.6 (figure 4).

**Table 3:** Risk factors for subtherapeutic NNRTI levels in 106 HIV positive Malawian Adults on Cart.

	Patients with therapeutic NNRTI concentrations n = 93	Patients with subtherapeutic NNRTI concentrations (range) n = 13	unadjusted OR	CI min	CI max	p value	Adjusted OR	CI min	CI max	p value
**Median age (range) [years]**	39 (18–64)	38 (22–69)	1.066	0.955	1.060	0.822	0.912	0.811	1.025	0.123
**Female gender [%]**	66 [76]	11 [58]	0.490	0.155	1.544	0.226	0.263	0.042	1.647	0.154
**Median completed years of schooling (range)**	7 (0–4)	7 (3–4)	0.846	0.457	1.567	0.595	1.303	0.552	3.076	0.546
**SRQ-20 score (range)**	5 (0–17)	5 (0–14)	1.061	0.938	1.200	0.349	1.090	0.852	1.394	0.493
**Median number of ART regimens (range)**	1 (1–17)	2 (1–17)	1.661	0.772	3.577	0.195	2.850	0.623	13.035	0.177
**Pill count (range) [%]**	99 (66–100)	98 (79–100)	**0.957**	**0.918**	**0.998**	**0.042**	**0.955**	**0.914**	**0.997**	**0.036**
**Number on EFV [%]**	18 [19%]	2 [15%]	1.136	0.287	4.500	0.857	0.575	0.58	5.753	0.638
**Number with MND/HAD [%]**	15 [16]	1 [8]	1.038	0.168	6.421	0.968	1.436	0.234	8.789	0.696

NNRTI: Non-nucleoside reverse transcriptase inhibitor, cART: combination anti-retroviral therapy, EFV: efavirenz, MND: mild neurocognitive disorder, HAD: HIV associated dementia, OR: Odds ratio, CI: confidence interval, min: minimum, max: maximum.

On linear regression, there was a direct correlation between age and plasma nevirapine concentrations (standardised beta coefficient 0.360, p value 0.014) and an indirect correlation with time since dose (standardised beta coefficient 0.360, p value 0.042). Linear regression for efavirenz concentrations was not performed due to the small number of patients.

Older age and higher SRQ-20 scores (indicating more symptoms of depression and anxiety) were associated with the presence of MND/HAD in unadjusted analyses. Only the SRQ-20 score remained significantly associated in the final multivariate model (table 4). Patients meeting criteria for ANI were significantly older and more likely to be female than those with no HAND (table 5). Subtherapeutic concentrations of NNRTI were not associated with the presence of HAND, MND and HAD combined or ANI.

**Table 4:** Risk factors for presence of MND/HAD in 106 HIV positive Malawian adults on cART.

	No HAND n = 32	MND/HAD n = 16	unadjusted OR	CI min	CI max	p value	Adjusted OR	CI min	CI max	p value
**Mean age in years (range)**	36.5 (22–59)	43 (29–69)	**1.089**	**1.019**	**1.164**	**0.012**	1.087	0.983	1.203	0.105
**Female gender (%)**	18 (56)	12 (75)	2.265	0.590	8.695	0.234	3.695	0.423	32.263	0.237
**Median completed years of schooling (range)**	7 (3–17)	7 (3–17)	0.780	0.360	1.692	0.530	0.858	0.247	2.984	0.810
**Median SRQ-20 score (range)**	2 (0–12)	8 (2–13)	**1.348**	**1.101**	**1.651**	**0.004**	**1.435**	**1.080**	**1.907**	**0.013**
**Median number of ART regimens (range)**	1 (1–3)	1.5 (1–3)	1.608	0.595	4.350	0.349	2.143	0.284	16.139	0.460
**Pill count (range) (%)**	99 (84–100)	98 (68–100)	1.007	0.913	1.112	0.883	1.035	0.893	1.200	0.645
**Number on EFV (%)**	5 (16)	5 (31)	1.300	0.266	6.352	0.746	0.122	0.005	2.699	0.183
**Number with subtherapeutic NNRTI concentrations (%)**	4 (13)	1 (6)	0.963	0.156	5.954	0.968	0.347	0.011	11.253	0.551

NNRTI: Non-nucleoside reverse transcriptase inhibitor, cART: combination anti-retroviral therapy, EFV: efavirenz, MND: mild neurocognitive disorder, HAD: HIV associated dementia, OR: Odds ratio, CI: confidence interval, min: minimum, max: maximum, HAND: HIV associated neurocognitive disorder.

**Table 5:** Risk factors for presence of ANI in 106 HIV positive Malawian adults on cART.

	No HAND n = 32	ANI n = 58	unadjusted OR	CI min	CI max	p value	Adjusted OR	CI min	CI max	p value
**Mean age in years (range)**	36.5 (22–59)	40.5 (18–71)	1.031	0.984	1.080	0.200	**1.068**	**1.007**	**1.133**	**0.028**
**Female gender [%]**	18 (56)	51 (88)	**3.104**	**1.219**	**7.907**	**0.018**	**4.281**	**1.394**	**13.143**	**0.011**
**Median completed years of schooling (range)**	7 (3–17)	7 (0–17)	0.732	0.460	1.165	0.188	0.922	0.495	1.714	0.796
**Median SRQ-20 score (range)**	2 (0–12)	4 (0–17)	1.120	1.002	1.252	0.046	1.065	0.919	1.234	0.402
**Median number of cART regimens (range)**	1 (1–3)	1 (1–3)	1.227	0.649	2.320	0.528	0.932	0.361	2.406	0.884
**Pill count (range) [%]**	98.8 (84.3–100.0)	99 (65.5–100.0)	0.984	0.939	1.030	0.490	1.001	0.944	1.060	0.986
**Number on EFV (%)**	5 (16)	10 (17)	1.380	0.443	4.295	0.579	0.937	0.198	4.426	0.934
**Number with subtherapeutic NNRTI concentrations (%)**	4 (13)	8 (14)	1.000	0.276	3.619	1.000	0.577	0.092	3.605	0.556

NNRTI: Non-nucleoside reverse transcriptase inhibitor, cART: combination anti-retroviral therapy, EFV: efavirenz, MND: mild neurocognitive disorder, HAD: HIV associated dementia, OR: Odds ratio, CI: confidence interval, min: minimum, max: maximum, HAND: HIV associated neurocognitive disorder.

Using linear regression to model variables associated with a higher GDS, only older age was significant (p = 0.04). Patients taking efavirenz had a significantly higher GDS than those taking nevirapine (0.615 vs 0.307, p<0.001), but this did not remain significant on multivariate analysis.

## Discussion

We found that in a population of adult Malawian ART patients with a high prevalence of HAND, plasma NNRTI concentrations were not lower in those with a HAND diagnosis. Having a subtherapeutic drug plasma concentration was neither associated with ANI nor with HAD/MND. In fact, subtherapeutic drug levels were found exclusively in patients with a GDS of less than 0.6, indicating normal neurocognition. The absence of subtherapeutic drug levels in patients with normal GDS scores suggests that the mainly milder abnormalities of neurocognition found in our patients do not affect drug adherence importantly in our setting. This may have multiple explanations, including the use of a guardian system, where each patient must have a guardian who goes through ART counselling with the patient and is encouraged to support their patient's medication adherence. Additionally, those with higher drug levels may experience neurotoxicity secondary to cART side effects [35]. In particular, high plasma levels of efavirenz are associated with an increase in neuropsychiatric disease [36], [37]. However the number of patients on efavirenz was small and we did not observe increased rates of HAND or higher GDS scores in this group compared to those taking nevirapine.

Fifteen percent of adult Malawian ART patients in our study had MND or HAD, which is comparable to studies reporting on the prevalence of HAND in developed countries [5], [10], [38], [39]. In sub-Saharan Africa, volunteers testing HIV negative at VCT clinics are routinely used as controls for HIV clinical and epidemiological studies. However, we found significant differences in educational attainment, occupation and likelihood of having anxiety or depression compared with HIV-positive patients. These are likely to have resulted in overestimation of patients who met criteria for ANI. The use of the IHDS to recruit patients on a 1∶1 basis may also have overestimated the true prevalence of HAND in our clinic.

Nevertheless, these are the first published normative values for neurocognitive testing in HIV negative Malawians, and our data are comparable to those obtained in a neuropsychological test battery in Uganda [38]. However, when taking the GDS to provide a measure based purely on neurocognitive performance, 16% of patients had a GDS of above 1 (representing mild neurocognitive impairment). Thus, it is not possible to conclude that our patients meeting the criteria for ANI had true HIV induced cognitive impairment. Some researchers have suggested the Frascati criteria potentially overestimate milder forms of HAND, and studies in sub-Saharan Africa have modified inclusion criteria to allow for this [5], [40]. Our estimates for MND or HAD are however more robust, since there was the requirement for mild to major functional impairment in addition to neuropsychological test performance. These estimates are consistent with other cohorts and we observed a skewed distribution of high z-scores in HIV positive patients indicating clearly impaired test performance. We suggest that in sub-Saharan Africa, normative data should ideally be obtained from a community based cohort instead of an HIV testing facility located in tertiary care. The high prevalence of symptoms of depression in our population is consistent with other literature reporting on depression and HAND [39], [41]. The presence of symptoms of depression has been suggested to be higher in older patients with HIV infection [42]. The fact that age and female gender were associated with ANI may be due to cultural and educational factors leading to sub-optimal performance on neurocognitive testing. However, evidence now supports a strong interaction between HIV and aging, where chronic HIV infection predisposes to conditions traditionally associated with aging, including neurocognitive impairment [43].

Other limitations to this study included a fairly small study population, which was due to the nature of intensive neuropsychological testing but limited power to detect small differences. NNRTI concentrations were chosen as a measure of long term adherence on the basis of their long plasma half-life, but due to the cross sectional nature of the study, we only had one measurement of plasma drug concentrations per patient. Although we did not have general access to HIV viral load testing, another objective measure of adherence, the five patients with the worst neurocognitive impairment who had a viral load test as part of their clinical assessment were all found to have undetectable plasma HIV-1 RNA. Because the neurocognitive battery was time consuming, we only performed a single assessment of functional impact using the Karnofsky Performance Score. It is possible that this score underestimated the functional impact in our study for cultural reasons as patients often seemed reluctant to admit to neurocognitive symptoms. In addition to challenges with neurocognitive classification, the aetiology of ANI also remains unclear. For instance, evidence is emerging to suggest that neurocognitive impairment in HIV positive patients may be mediated by effects on endothelial damage rather than direct CNS effects of the virus [44], [45]. Although we took full medical histories from all patients, it is possible that other co-morbidities may have been involved in neurocognitive impairment in HIV. This normative data will provide a foundation for future neurocognitive studies and clinical neurocognitive outcomes in Malawi, both in HIV and in other diseases with neurocognitive impact.

In conclusion, we found that in adult Malawian ART patients a diagnosis of symptomatic neurocognitive impairment was not associated with poor adherence as measured by subtherapeutic plasma NNRTI concentrations. To our knowledge, no other studies have correlated HAND with adherence in sub-Saharan Africa, defined by plasma drug concentrations. We found that that pill count and drug level were associated, underlining the reliability of the adherence measures used. In order to improve the diagnosis of patients with clinically significant HAND in our region, future research should build on this foundation to gather more robust normative data. The clinical relevance of ANI in sub-Saharan Africa needs to be determined in further studies that should address the wider impact of HAND and use a larger study population.
